# The Largest Reported Giant Ascending Aortic Aneurysm Presented with Superior Vena Cava Syndrome

**DOI:** 10.21470/1678-9741-2019-0151

**Published:** 2020

**Authors:** Murat Bicer, Ahmet Yuksel, Iris Irem Kan

**Affiliations:** 1 Department of Cardiovascular Surgery, Uludag University Faculty of Medicine, Bursa, Turkey.; 2 Department of Cardiovascular Surgery, Abant Izzet Baysal University Faculty of Medicine, Bolu, Turkey.

**Keywords:** Ascending Aortic Aneurysm, Giant, Surgical Treatment

## Abstract

Giant ascending aortic aneurysm is a rare condition. In this paper, we present an uncommon case of giant ascending aortic aneurysm with a maximal diameter of 14 cm in a 77-year-old woman presenting with unusual symptoms. The patient underwent a successful surgery involving ascending aortic replacement, and was discharged without any complication. After discharge, she was followed regularly and no major problem was observed in her control visits. To the best of our knowledge, our case is the largest ascending aortic aneurysm reported to date in the existing literature.

**Table:** 

Abbreviations, acronyms & symbols	
AAA	= Ascending aortic aneurysm
CT	= Computed tomography
MR	= Magnetic resonance
TEE	= Transesophageal echocardiography

## INTRODUCTION

Giant ascending aortic aneurysm (AAA) is a rarely encountered condition, and few cases have been reported in the literature. Appropriate and timely surgical management of patients with giant AAA often provides the only chance in order to prolong the life span. Life expectancy without surgical treatment for such patients is very low, and they usually die from either the rupture of aneurysm or decompensation of organs and systems^[1,2]^. We herein report a case of giant AAA measured 14 cm in its maximum diameter which was successfully treated with open surgical repair. To the best of our knowledge, this case is the largest reported case of giant AAA in the existing literature.

## CASE REPORT

**Fig. 1:**
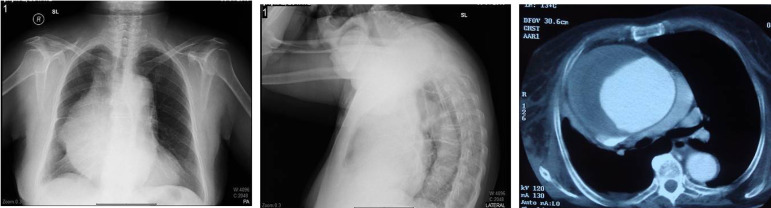
(A and B) – Aneurysm appearance on posteroanterior and lateral chest radiography. (C) – Aneurysm appearance on computed tomography scan.

**Fig. 2:**
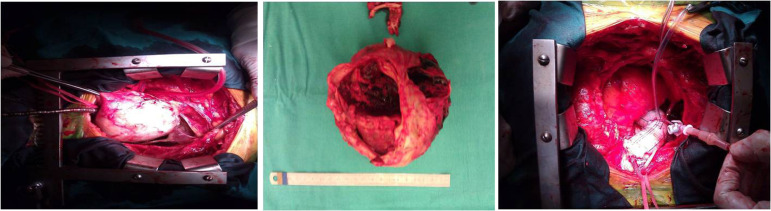
(A) – Giant ascending aortic aneurysm in the pericardial cavity. (B) – Excised aneurysm. (C) – Aorta with 32 mm Dacron polyester fabric graft.

A 77-year-old woman was admitted to our hospital with atypical symptoms. She had dyspnea when she lay on her back or in supine position. Also, she complained of the symptoms of superior vena cava syndrome in the mornings but in hours her symptoms were relieved. The symptoms and signs of superior vena cava syndrome in this case were intermittent cough and dysphagia, facial and neck swelling, bilateral jugular venous distention and collateral venous circulation. In her medical history, there was no history of previous surgical intervention and she only had the existing diagnosis of hypertension. Physical examination demonstrated a slight decrease in breath sounds over the right hemithorax. Chest radiography showed widened mediastinum, increased cardiothoracic ratio, deviation of the trachea, and convexity of the right superior mediastinum (Figure 1A and 1B). A contrast enhanced computed tomography (CT) scan confirmed the existence of ascending aortic aneurysm which compressed the superior vena cava, and its maximum diameter was 14 cm (Figure 1C). It was decided that a prompt open surgical repair of this giant AAA was the most appropriate treatment option, due to the existence of high risk of rupture of aneurysm, hemorrhagic shock and death. After informing about the operation and obtaining the informed surgical consent form from the patient, she was transferred to the operating room and operated on in supine position under general anesthesia. Right common femoral vessels were exposed and cannulated in order to establish cardiopulmonary bypass, and then a median sternotomy was performed. A giant AAA was occupying most of the space in the pericardial cavity (Figure 2A). The aorta was cross clamped across the aortic arch. Under normothermic condition, an aortotomy was made and we used selective cardioplegia directly into both coronary ostia. Afterwards, the ascending aorta was excised (Figure 2B), and the aneurysm was replaced with a size 32 mm Dacron polyester fabric graft as supracoronary by using 3-0 prolene sutures supported by teﬂon strips, without performing any aortic valve and coronary artery procedures (Figure 2C). Weaning from cardiopulmonary bypass and postoperative course were uneventful. The patient was discharged 6 days after the operation. She was followed up regularly, and no major problem was observed in her control visits.

## DISCUSSION

Ascending aortic aneurysms (AAAs) are the most common subtype of thoracic aortic aneurysms, and about 60% of thoracic aortic aneurysms involve the ascending aorta or aortic root^[3]^. Cystic medial necrosis is the most common pathological reason of aortic aneurysms. While the most common etiological reason of AAAs in older patients is arteriosclerotic degeneration, Marfan syndrome or bicuspid aortic valve that may be associated with aortic pathology are the most common reasons in younger patients. Other etiological reasons of AAAs are trauma, aortic pseudoaneurysms, aortic dissection and several forms of vasculitic diseases such as Takayasu arteritis and giant cell arteritis. The majority of patients with AAA is asymptomatic, and is diagnosed with incidental ﬁndings on radiological imaging modalities. However, larger aneurysms may have a varied clinical presentation which usually results from compression or eroding of surrounding structures and organs such as chest wall, cardiac chambers, pulmonary artery, trachea and oesophagus^[4]^. They may lead to chest or back pain and hoarseness secondary to recurrent laryngeal nerve paralysis. Similarly to our case, they may also be presented as a rare reason of superior vena cava syndrome^[1]^. In addition to these, the large AAAs may be even presented by aortic dissection or rupture into pericardial cavity leading to cardiac tamponade, circulatory collapse and death.

It may be benefited from several imaging tools in order to establish of diagnosis of AAA. Chest radiography may demonstrate a widened, convex right superior mediastinum, a deviation of the trachea because of mass effect, and a shadow to the right of the cardiac silhouette. Transthoracic echocardiography shows the function of aortic valve and proximal ascending aortic structure. It may aid to detect aortic regurgitation and AAA, however its sensitivity and specificity are lower than transesophageal echocardiography (TEE). TEE, contrast enhanced CT and magnetic resonance (MR) angiography have nowadays become commonly used imaging modalities in order to confirm of diagnosis of AAA and precise its dimensions. Among them, contrast enhanced CT scan (particularly multi-detector CT mode) is the most popular radiological modality in the assessment of an AAA, as CT provides the best quality method for a detailed analysis of the aneurysmal morphology^[5]^.

In general, surgical indications of AAA are based on size and growth rate of aneurysm, and the existence of symptoms. American College of Cardiology, American Heart Association and European Society of Cardiology guidelines^[6,7]^ have recommended the surgical repair for all symptomatic AAAs (ruptured, associated with dissection, causing pain) regardless of aneurysm size. For asymptomatic patients, these guidelines have recommended the surgical repair in the existence of degenerative thoracic aneurysm, chronic aortic dissection, intramural hematoma, penetrating atherosclerotic ulcer, mycotic aneurysm or pseudoaneurysm, and for patients whom the ascending aorta or aortic sinus diameter is 5.5 cm or greater. On the other hand, asymptomatic patients with Marfan syndrome or other genetically mediated diseases such as Ehlers-Danlos syndrome, Turner syndrome, bicuspid aortic valve and familial thoracic aortic aneurysm should be performed surgery at smaller diameters of 4.0-5.0 cm, depending on the condition, in order to avoid acute aortic dissection or rupture. Additionally, rapid expansion of an AAA is considered as another surgical indication.

Giant AAA is defined as an aneurysm with maximal diameter greater than 10 cm^[8]^. Surgical management of a giant AAA is challenging since it poses some complication risks such as bleeding, end organ ischemia, underlying disease and staged repairs^[5,9,10]^. During the operation, performing median sternotomy may lead to aortic injury since the aneurysmal wall is very close to sternum. Therefore, the exploration and cannulation of femoral vessels for the establishment of cardiopulmonary bypass before the median sternotomy are recommended^[2,8]^. Antegrade and retrograde cerebral perfusion for the protection of neurocerebral functions is important, which prevent neurologic adverse events. Furthermore, deep hypothermia and circulatory arrest may be also preferred during the surgery; however we did not prefer deep hypothermia and circulatory arrest in our case, and did not observe any complication during perioperative period.

In this presented case, we successfully performed a prompt open surgical repair of a giant AAA, and obtained a satisfactory result without morbidity and mortality during postoperative period. To the best of our knowledge, our case is the largest AAA reported to date in the existing literature.

## CONCLUSION

Giant AAA is a rare entity and sometimes leads to unusual symptoms. Its surgical treatment remains the standard approach regardless of aneurysm size and age of patient, and surgical outcomes are mostly satisfactory.

**Table:** 

Author's roles & responsibilities	
MB	Substantial contributions to the conception or design of the work; or the acquisition, analysis, or interpretation of data for the work; drafting the work or revising it critically for important intellectual content; final approval of the version to be published
AY	Substantial contributions to the conception or design of the work; or the acquisition, analysis, or interpretation of data for the work; drafting the work or revising it critically for important intellectual content; final approval of the version to be published
IIK	Substantial contributions to the conception or design of the work; or the acquisition, analysis, or interpretation of data for the work; drafting the work or revising it critically for important intellectual content; final approval of the version to be published
